# The New Mexican Remedy for Dysentery

**Published:** 1894-11

**Authors:** A. N. Perkins

**Affiliations:** Sabine Pass, Texas


### The New Mexican Remedy for Dysentery.

SABINE PASS, TEXAS, October 20, 1894.

*Editor Texas Medical Journal:*

Less than a year ago Dr. Knox, of Gonzales, published in the TEXAS MEDICAL JOURNAL a paper on the Mexican remedy for chronic diarrhœa, *chaparro amargoso.* He recommended it very highly, and testified to the wonderful effect on himself.

At the time Dr. Knox's paper was published, there was a respectable citizen of this county who had been suffering with chronic diarrhœa for several years, and had despaired of ever getting well. His disease had baffled the skill of the doctors. When I read Dr. Knox's paper, I determined to give our unfortunate citizen the benefit of the remedy. I immediately wrote to Dr. Knox to send me some of it. He kindly did so, and I gave it to the afflicted man, and told him how to take it. About one month from that time, I met him in the street, and he joyfully informed me that he was as well as he ever was in his life. I never saw a happier man than he appeared to be. He remarked, "that medicine is worth its weight in gold."

Six weeks ago I was asked to see a lady. She was almost a skeleton from the effects of chronic diarrhœa. She told me that she had been under the treatment of several prominent physicians, but had only been temporarily benefitted. She had little faith of ever being cured. I had procured a fluid extract of the chaparro amargoso, made by the distinguished druggists, Sharp & Dohme, of Baltimore. I gave her four ounces of the fluid extract, and told her to begin with half a teaspoonful, and increase to one teaspoonful, three times daily. She informs me that she considered herself entirely well by the time she had taken half of the medicine. She is now the picture of perfect health.

This is the extent of my experience with the new remedy. It certainly acted wonderfully in these two cases. There are, of course, cases of chronic diarrhœa where disease has created organic lesions of such a nature that nothing can ever restore the bowels to their normal condition. The chaparro will fail in cases of this kind. It is a valuable remedy.

A. N. PERKINS, M. D.

---

### The Transactions: A Correction.

AUSTIN, TEXAS, October, 1894.

*Editor Texas Medical Journal:*

